# The Exposure Assessment in Current Time Study: Implementation, Feasibility, and Acceptability of Real-Time Data Collection in a Community Cohort of Illicit Drug Users

**DOI:** 10.1155/2013/594671

**Published:** 2013-11-06

**Authors:** Gregory D. Kirk, Beth S. Linas, Ryan P. Westergaard, Damani Piggott, Robert C. Bollinger, Larry W. Chang, Andrew Genz

**Affiliations:** ^1^Department of Epidemiology, Johns Hopkins University, Baltimore, MD 21205, USA; ^2^Department of Medicine, Johns Hopkins University, Baltimore, MD 21205, USA; ^3^Department of Medicine and Population Health, University of Wisconsin-Madison, Madison, WI 53792, USA

## Abstract

*Objective*. We describe the study design and evaluate the implementation, feasibility, and acceptability of an ecological momentary assessment (EMA) study of illicit drug users. *Design*. Four sequential field trials targeting observation of 30 individuals followed for a four week period. *Participants*. Participants were recruited from an ongoing community-cohort of current or former injection drug users. Of 113 individuals enrolled, 109 completed study procedures during four trials conducted from November 2008 to May 2013. *Methods*. Hand-held electronic diaries used in the initial trials were transitioned to a smartphone platform for the final trial with identical data collection. Random-prompts delivered five times daily assessed participant location, activity, mood, and social context. Event-contingent data collection involved participant self-reports of illicit drug use and craving. *Main Outcome Measures*. Feasibility measures included participant retention, days of followup, random-prompt response rates, and device loss rate. Acceptability was evaluated from an end-of-trial questionnaire. Sociodemographic, behavioral, clinical, and trial characteristics were evaluated as correlates of weekly random-prompt response rates ≥80% using logistic regression with generalized estimating equations. *Results*. Study participants were a median of 48.5 years old, 90% African American, 52% male, and 59% HIV-infected with limited income and educational attainment. During a median followup of 28 days, 78% of 11,181 random-prompts delivered were answered (mean of 2.8 responses daily), while 2,798 participant-initiated events were reported (30% drug use events; 70% craving events). Self-reported acceptability to study procedures was uniformly favorable. Device loss was rare (only 1 lost device every 190 person-days of observation). Higher educational attainment was consistently associated with a higher response rate to random-prompts, while an association of HIV infection with lower response rates was not observed after accounting for differences in trial recruitment procedures. *Conclusion*. Near real-time EMA data collection in the field is feasible and acceptable among community-dwelling illicit drug users. These data provide the basis for future studies of EMA-informed interventions to prevent drug relapse and improve HIV treatment outcomes in this population.

## 1. Introduction

Optimal HIV care requires prompt identification of HIV infection, linkage to HIV care, prolonged engagement in HIV care with regular attendance at appointments, and high levels of adherence to antiretroviral regimens in order to achieve viral suppression [1]. Illicit drug use can have negative impacts at each stage of this HIV care continuum [2, 3]. Despite substantial research, identification of the proximate predictors of relapse to illicit drug use, nonadherence to HIV medications, or disengagement with primary HIV care among drug users remains elusive. Through data collection in near real time among persons going about their daily lives, ecological momentary assessment (EMA) methods attempt to sample persons' real-life experiences and may capture the contextual factors which precede events such as drug use or nonadherence [4, 5].

The promise of mobile health (mHealth) technologies for strengthening HIV care delivery has been acknowledged by the United Nations Joint Programme on HIV/AIDS (UNAIDS) strategic plan [6]. Text messages have been effectively utilized to improve attendance at clinic appointments, promote adherence to antiretroviral therapy (ART), and increase rates of viral suppression [7–14]. However, most of the smartphone applications developed for use in HIV care settings, have not been widely used or reviewed favorably [15]. Further, few studies have been directed at substance-using HIV-infected populations in resource-intensive countries or have utilized EMA approaches. In non-HIV-infected populations in the USA, EMA studies centered in drug treatment settings have demonstrated a strong correlation between tobacco, cocaine, and heroin craving, and related urine-verified periods of cocaine abstinence to negative moods, stress levels, and to daily patterns of recreation and work activity [5, 16–18].

In response to the limited application of EMA approaches to out-of-treatment, high-risk populations, we sought to develop EMA methods for near real-time characterization of illicit drug use occurring in users' natural environments. In this paper, we describe the study design and participants of the EXposure Assessment in Current Time (EXACT) study. Further, we characterize implementation barriers and examine the feasibility and acceptability of using intensive EMA data collection methods among community-dwelling illicit drug users with or at-risk for HIV infection.

## 2. Methods 

### 2.1. Study Participants

**Figure 1:**
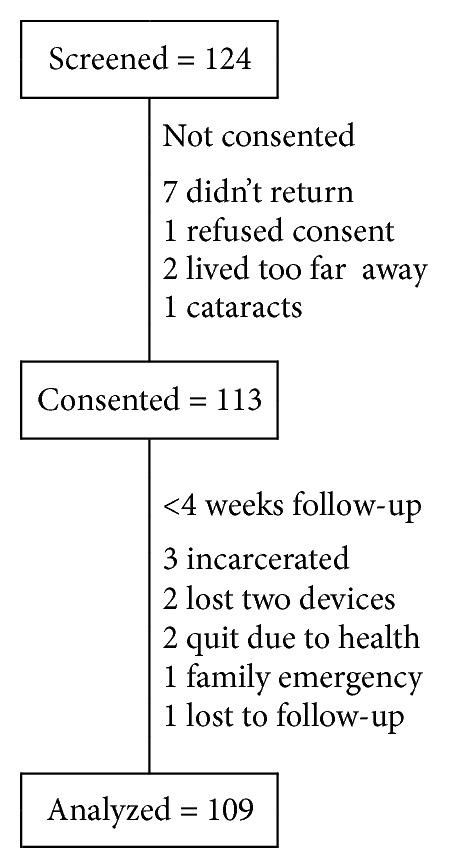
EXACT Participants flow diagram.

EXposure Assessment in Current Time (EXACT) study participants were recruited from the AIDS Linked to the IntraVenous Experience (ALIVE) study, an ongoing, community-recruited, observational cohort of persons with a history of injecting drugs in Baltimore, MD [19]. The ALIVE cohort is community-based rather than clinic-based, thereby reducing selection bias toward persons seeking or accessing care for drug use or for HIV infection. From November 2008 through May 2013, four successive field trials were conducted. Each trial followed approximately 30 participants for four weeks. ALIVE cohort participants attend regularly scheduled appointments for semiannual study visits, thus, allowing a carefully regulated environment in which to invite participation in this substudy. Eligibility criteria for EXACT included current enrollment in ALIVE, residence in Baltimore City, and the ability to understand and follow directions on a personal digital assistant (PDA) or mobile phone. Individuals were excluded if they had any medical condition that would prevent them from operating the hand held device (e.g., visual or hearing impairment) or if they failed to attend the screening appointment where they were trained how to use the device.  Figure 1 shows a flow diagram of recruitment and retention in the study. Of persons that were referred, attended a screening visit, and found to meet eligibility criteria, and enrolled, 93% of participants completed the four weekly study visits. In successive trials, the specific inclusion criteria regarding drug use and HIV status were varied slightly. In Trial 1, selection was made to balance the numbers of participants that reported recent heroin or cocaine use with those that were not currently using drugs. In Trial 2, all participants reported heroin or cocaine use within the prior three months. While HIV status was not a recruitment criterion in the first two trials, Trials 3 and 4 included only HIV-infected participants who reported recent heroin or cocaine use, with preference for those using on average at least three times weekly in the past month.

The Johns Hopkins School of Public Health approved the study protocol. An existing Certificate of Confidentiality through the National Institute on Drug Abuse was amended to include EXACT study procedures. All participants provided written informed consent. Participants in the EXACT study were informed that involvement (or noninvolvement) in EXACT would in no way affect their participation in ALIVE.

Funding for the study was provided through the National Institute on Drug Abuse as part of the Network on Exposure to Psychosocial Stress and Addictive Substances, a component of the NIH's Genes, Environment and Health Initiative [20].

### 2.2. Study Procedures

Current EMA methodology generally employs electronic diaries or mobile devices to collect data on both a random and an event-contingent schedule. For random-prompt data collection, EXACT participants responded to an alarm that sounded at a set number of randomly spaced times throughout the day by answering a questionnaire on the device. For event-contingent data collection, participants self-initiated a questionnaire in close temporal proximity to an event of interest (e.g., drug use or craving); participants were required to confirm that the event occurred within the last 30 minutes. 

EXACT participants were provided a hand-held electronic device to carry for four weeks for real-time data collection. For Trials 1–3, participants were provided PDAs (Palm Z22, Palm, Inc., Sunnyvale, CA, USA) running applications developed using Satellite Forms software (http://www.satelliteforms.net/). To reduce the street value of handheld devices, all PDA programs were disabled except for study-required applications. In Trial 4, participants were provided an Android smartphone (Motorola Droid X2), running the electronic Mobile Open-source Comprehensive Health Application (eMOCHA), developed at Johns Hopkins School of Medicine [21]. Previously, eMOCHA had been used to support community health workers in resource-limited settings that were heavily impacted by HIV [22] and was modified specifically for this study. Other functions of the smartphones were not disabled, although superfluous applications such as games were uninstalled whenever possible. In order to collect real-time geolocation data, participants in Trials 2 and 3 carried a global positioning system (GPS) unit (QSTARZ BT-Q1000X, Taipei, Taiwan) which recorded their latitude and longitude every five minutes or if they moved more than five yards. In Trial 4, GPS data collection was obtained from the geolocation tracking system of the smartphone.

**Figure 2:**
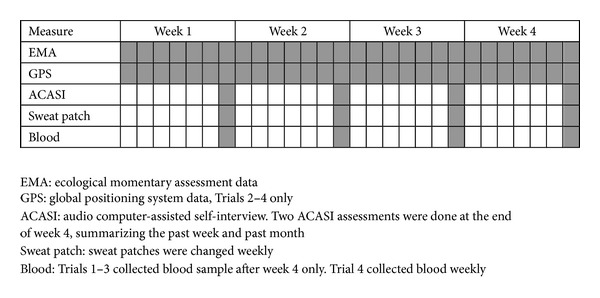
EXACT data collection procedures.

Aside from the device provided, the study procedures and data collection in the trials were essentially identical (Figure 2). Random-prompts were delivered four times daily between 9:00 am and 9:00 pm, with a fifth prompt, set for 9:01 pm each day (end-of-day questionnaire). Alarms would repeat every three minutes up to a maximum of five times. Participants were required to complete the questionnaire within 15 minutes or the prompt was considered missed. Twice each week, in Trials 1–3 using the PDA, participants returned to the clinic for device maintenance and to upload their data to a secure server. All participant data was then erased from both the PDA and GPS units. In Trial 4, EMA and GPS data were continuously transmitted in encrypted fashion to the eMOCHA secure server for storage.

EMA studies provide the ability to collect data in participant's natural settings. EMA questions were developed to assess the social, psychosocial, physical, and activity context of the participants' current environment. Survey instruments were adapted from prior EMA studies conducted by collaborators working with drug using populations [5, 16, 23]. Participants were asked to self-report each time they either craved (but refrained from using) or used heroin or cocaine; these self-initiated responses constitute the event-contingent data collection.

In all trials, at the end of each week, participants answered an audio-computer assisted standardized interview (ACASI)-based version of the end-of-day questionnaire modified to reflect activities, behavior and events during the prior week. Similarly, at the conclusion of each trial, participants completed an ACASI-based questionnaire designed to reflect the entire four week study period. Hair and/or sweat patch samples were collected weekly for measurement of illicit substances, and blood samples were collected at the end of the trial period to be tested for potential biomarkers of psychosocial stress. Participants received remuneration for attendance at study visits, for providing adequate responses to weekly random-prompts, and for returning devices upon study completion. Participants were informed at entry that loss of two study devices would result in dismissal from the study.

### 2.3. Data Analysis

Baseline sociodemographic (e.g., age, sex, race, education, marital status, employment, income, homelessness, and health insurance status), behavioral (e.g., self-reported alcohol, tobacco, and illicit drug use), and clinical (e.g., HIV/antiretroviral therapy status, CD4 T-cell count, and HIV RNA levels) characteristics were obtained from the existing ALIVE database. Depressive symptoms were assessed using the Center for Epidemiologic Studies Depression Scale (CES-D) for the 6 months prior to EXACT study entry.

Characteristics of participants, days of followup, random-prompt response rates (overall and by week), and device loss rate were examined by trial number. Using a response rate of ≥80% to weekly random-prompts as a dichotomous outcome variable, logistic regression models with generalized estimating equations (GEE) were evaluated to identify the sociodemographic, behavioral, clinical, and trial-related correlates of higher response rates. Analyses were performed using Stata statistical software (version 11). 

## 3. Results

We analyzed data from 109 participants who had at least one week of follow-up. Nine participants were followed less than the full four weeks possible (Figure 1). For 109 EXACT participants with evaluable data, the median age was 48.5 years, 90% were African American, and 52% were male (Table 1). A majority of participants had not completed high school, had never been married, and had an annual formal income of <$5000. At study entry, a majority of participants reported recent substance use, including the consumption of cigarettes (83%), alcohol (65%), and heroin or cocaine (61%). Among the 59% of participants that were HIV infected, the median CD4 cell count was 361 cells and 55% had an HIV RNA level >500 copies/mL.

Overall, the 109 participants provided 3,047 days of observation (median of 28 days per person; IQR 26–29) with little variability between trials (Table 2). A total of 11,181 random-prompts were delivered, which represented 78% of planned prompts. In particular, delivery of random-prompts was lower in Trial 3 (60%) due to technical problems. Delivery was not completed and data were lost if the Palm devices reset accidentally (a common occurrence) or if the battery ran out. In Trial 4 using the eMOCHA smartphone platform, random-prompt delivery was notably more efficient at 98%. 

The overall proportion of random-prompts responded to was 78%, which translated to an average of 2.84 random-prompt responses per day. The response rate was relatively consistent in Trials 1 and 2 but was lower in trials which targeted more intense, recent drug users (Trials 3 and 4). Despite the lower response proportion in Trial 4, the more efficient delivery of prompts by smartphone translated into a greater average number of prompts answered daily (3.45 compared to the overall estimate of 2.84). Problems in the PDA studies including technical problems, battery outage, and device loss or damage resulted in the loss of several days' data collection. Further, the data collection software required that the data be manually uploaded using a USB cable from the PDA to the computer where the database was located. The eMOCHA software eliminated this requirement by transmitting the data wirelessly and securely to a server approximately every 15 minutes. 

**Figure 3:**
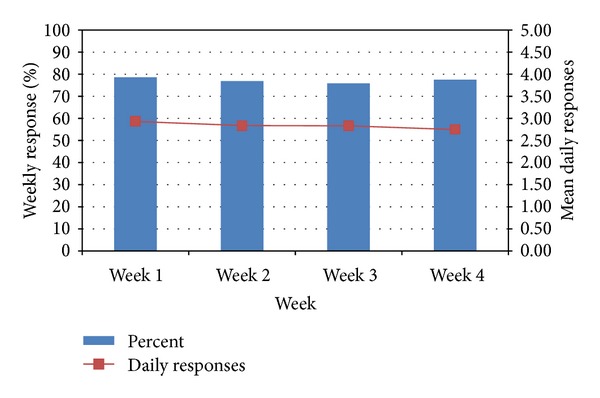
Responses to random-prompts by week of trial (% answered; daily mean number of responses).

Recognizing concerns that exhaustion with responding to device prompts may occur, we examined the response rates and average daily number of prompts answered by week of trial (Figure 3). Overall, there was no meaningful difference in either the response proportion or average number of responses between weeks. Similar to the overall four-week response rate data, we did observe tapering of response rates in Trial 4 in later weeks, although the average number of prompts answered daily remained higher than other trials. 

Regarding event-contingent data, there were 2,798 participant-initiated events reported, representing 70% drug craving and 30% drug use events (Table 2). Drug craving events appeared to be reported most commonly in Trial 1, while drug use events were reported more commonly in Trials 1 and 3. 

Of 201 devices issued to participants, 15 were lost or damaged which translates to the loss of a device once every 190 days of observation. Only two participants (1.8%) were excluded from continuing in the study because they lost two devices. Monetary incentives were provided for active participation, which averaged about $15 per day of observation. 

In assessment of participant acceptability (Table 3), favorable responses were uniformly reported regarding ease of use of the devices (98% reported easy or very easy), the burden of reporting (93% reported just right or not enough), the understandability of the questions (89% reported most or all make sense), and confidence in privacy protections (91% reported mostly or extremely confident). Twenty-nine percent of respondents agreed that carrying the devices made them behave differently.

To understand what factors may contribute to higher levels of active participation in EMA studies, we examined correlates of responding to ≥80% of weekly random-prompts (Table 4). Of 405 total weeks included in the analysis, 199 (49%) were weeks with ≥80% response rate. Among sociodemographic variables, educational attainment was strongly and consistently associated with higher response rates; there was no association with age, gender, race, or homelessness. Neither recent history of heroin or cocaine use prior to study entry nor heroin or cocaine use reported by EMA during the same week was associated with decreased odds of a ≥80% response rate. 

Trials 3 and 4 were associated with lower responses (Table 4, Model B). Because of selection criteria for Trials 3 and 4 in which recruitment targeted only HIV-infected persons with attempts to enrich for more active drug users, the variables of HIV status and trial were related to each other resulting in collinearity in the models. Therefore, it is difficult to establish what factors explain the lower responses. When not accounting for trial characteristics (Table 4, Model C), we observed a dose-response association with increasing odds of lower responses in HIV-infected persons with undetectable HIV RNA levels and even poorer responses in those persons with detectable viral load in comparison to HIV-uninfected persons. However, after accounting for trial characteristics (Table 4, Model D), HIV infection was no longer associated with poorer responses. 

## 4. Discussion

Illicit drug users are considered a challenging population to identify, recruit, and retain in epidemiological, behavioral, or clinical research. Consequently, many issues of primary concern to this population remain poorly understood, such as why some active drug users are able to maintain cessation and do well on HIV treatment while others have difficulty with drug relapse, ART nonadherence, and virological failure. By employing multiple methods to maximize retention, including strong rapport among clinic staff and participants, mailed appointment reminders, phone calls, tracing through contacts, street tracing, transportation vouchers, and minimal financial remuneration for participation, the ALIVE study has demonstrated the ability to successfully follow an IDU population over >25 years in a design incorporating study visits conducted every six months. Further, ALIVE represents a community-recruited cohort following IDUs outside of the provision of clinical care in research clinics situated in residential and commercial sections of East Baltimore rather than physically colocated in buildings of the Johns Hopkins Medical Institutions. Despite these successes, there clearly is potential for extremely valuable information on the proximate influences of drug users' behavior to be gained by refining the data collection window to minutes instead of months and of locating data collection to occur wherever persons may be, rather than relying on retrospective reporting in a formal clinic setting. The primary objective of the EXACT study was to develop EMA methods for real-time characterization of illicit drug use occurring in users' natural environments.

In this paper, we provide strong data supporting the feasibility and acceptability of using EMA methods for data collection among illicit drug using populations. Our recruitment and referral process from ALIVE was highly efficient. With inclusion of a dedicated screening visit, which served to ensure attendance at an additional study visit prior to enrollment and to gauge potential participants comfort level with the technology, we had a 93% rate of study completion. During the process of implementing the study, we were frequently met with healthy skepticism from other researchers regarding the likelihood of success in meeting our objectives. Our study protocol included 5 daily random-prompts, which represents a higher participant burden than many EMA studies. A recurring question from colleagues and IRB reviewers was whether illicit drug users would really answer all those questions. Overall, EXACT participants answered 78% of random-prompts, a response rate comparable to EMA studies performed using similar technologies in varied settings. Prior EMA studies in diverse populations including illicit drug users, chronic pain patients, and smoking cessation or obesity intervention participants achieved response rates to random-prompt surveys ranging from 70–80% [5, 24–26]. In questioning upon completion of the study, participants were very clear in reporting that study procedures were not overly burdensome. Although we did not conduct formal qualitative evaluations, our informal debriefs were consistent with our quantitative assessment of participant acceptability indicating that participants held strongly positive sentiments regarding the study procedures and their participation. 

We examined correlates of higher response rates and found that higher educational level was a strong and consistent predictor, while age, race, gender, and income level were not associated. Our study population had low overall educational attainment with only 41% completing high school or equivalency. It is unclear whether this association represents more ease or familiarity with the technology among more educated persons or whether education status is a surrogate marker for other factors which may facilitate high responses. In a recent survey conducted in ALIVE, we found that participants with greater education reported increased ownership of smartphones and an increased willingness to receive health-related communications through various technologies. Educational attainment is strongly associated with health literacy, defined as the ability to obtain, process, and understand the basic health information and services needed to make appropriate health decisions [27, 28]. These data highlight the need for mHealth interventions to be sensitive to the educational and health literacy levels of participants. Technologically-based platforms have significant potential to address the health needs of low health literacy individuals [29]. Formal evaluation of health and/or communication technology literacy may be appropriate with the provision of additional training and monitoring to persons with lower literacy and efforts focused on developing literacy-adapted interventions. Although underdeveloped to date, the concept of eHealth literacy merits further investigation, including the need for development of standardized and valid scales for its measurement [30].

We provided modest weekly incentives to participants for providing more complete responses to EMA questioning. Our lack of association of response rates with income and the finding that higher educational attainment (which would be expected to be associated with higher income) were associated with higher responses provides indirect evidence suggesting that the level of incentives was not coercive to vulnerable participants with limited income. Future studies will need to examine further the efficacy and ethics of providing incentives for improving EMA responses and to establish what type and level of incentives may be most appropriate. One EMA study has supplemented response-weighted incentives with weekly sessions between participants and research staff to review their individual response rates and emphasize compliance to obtain even higher levels of response [31]. 

In addition to higher educational attainment, our analysis of response rates to the random-prompts indicated that the later trials that selectively recruited HIV-infected participants with more intense recent substance use had lower response rates. This raises concern that the targeted population of out-of-care HIV-infected persons with detectable viremia and active illicit drug use may be less likely to actively participate in similar studies or interventions. However, it should be noted that after accounting for the differential recruitment criteria between trials, the association with HIV infection was no longer observed. Further, the mean number of daily responses was actually higher among HIV-infected participants. Ultimately, larger and longer trials will likely be needed to refine how those populations at greatest risk will engage and respond to EMA studies. 

Another challenge to using technological devices among drug user populations is concerned that participants might simply sell the devices. To mitigate this potential problem, we clearly delineated to participants during screening and enrollment procedures that the loss of two devices (whether reported as damaged, lost, or stolen) would result in their exclusion from the study. Further, we provided a small incentive for return of the devices upon completion of the study. Remarkably, only two persons (representing <2% of all participants) were excluded for lost devices. Overall, we averaged only one lost device per every 190 days of observation, which we feel represents an effective utilization of these technologies. We acknowledge that the street value for the PDAs is less than for the smartphones; however, in our limited data, there was not a substantial difference in the device loss rate between trials using the different devices. While the PDAs had nonstudy features locked out, we did not implement similar restrictions with the smartphones. Current technologies allow the remote inactivation of smartphones if they are stolen or misplaced which could represent an important deterrent, although this capability was not disclosed to participants nor employed in our study. Contemporary mHealth technologies are evolving at a rapid pace. In our study, the PDA model we used at study initiation was no longer being manufactured after 10 months of study recruitment and was largely obsolete by the date of study completion. Rapid transitions in technology, therefore, represent a significant barrier to successful implementation of mHealth programs, often limiting the interpretability and applicability of data from these studies. Our smartphone platform resulted in more efficient delivery, a higher average number of daily responses, and provided automatic data transfer which both enhanced security and minimized data loss. In moving forward with advancing technology, alternative approaches to trial design must be considered, including designs fitted to the stage of technology or intervention development and adaptive trials that allow for evolving technologies or intervention optimization [32]. 

In addition to the challenges described above, our study had other limitations. In these field trials designed to provide information on feasibility and acceptability, we followed a limited number of persons for approximately four weeks. It has been well recognized that many intensive data collection interventions may plateau or reach exhaustion within weeks or months, diminishing the level of participation and success of these strategies. Importantly, in our examination of weekly response rates, we did not find evidence for exhaustion. In the future, the broader goal of EMA studies would be to provide longer-term monitoring or intervention through trials that extend beyond our limited time frame. Despite our study being nested within an IDU cohort, many participants were not actively using drugs. In these early studies, we selected for a range of drug use intensity and the resulting cohort overall would not be considered heavy users. Therefore, our findings may not extend to drug users with more intense drug use patterns.

Finally, EXACT participants were largely African American from a single urban center, raising concerns regarding the generalizability of our findings. However, our IDU population represents among the most disadvantaged and vulnerable drug using populations, and our demonstration of successful implementation of EMA methods in this population bodes well for efforts to apply these methods to less challenging populations. Further, the aging, African American IDU epidemic in Baltimore is similar to many other urban IDU epidemics in the USA, from Newark to St Louis to Detroit. It is these populations that are at greatest risk for limited access to appropriate drug treatment or HIV care that are most in need of innovative and tailored interventions to improve outcomes. 

In conclusion, findings from the EXACT study demonstrate that EMA methods are feasible and acceptable approaches for data collection among illicit drug users. As the next important step, interventions that leverage the predictive value of intensive EMA data collection to allow real-time tailored interventions in drug users' natural environments (i.e., ecological momentary interventions [EMI]) will need to be developed and rigorously evaluated. The translation of intensive EMA data into tailored EMI holds promise for improving our understanding and our ability to reduce relapse, improve engagement in care, enhance ART adherence, and to intervene on other difficult issues surrounding drug use and HIV care.

## Figures and Tables

**Table 1:** Characteristics of EXACT participants by trial.

Characteristic	All trials (*N* = 109)	Trial 1 (*N* = 31)	Trial 2 (*N* = 28)	Trial 3 (*N* = 28)	Trial 4 (*N* = 22)
Sociodemographic variables					
Median age, yrs (IQR)	48.5 (43.3–52.9)	48.5 (41.8–52.3)	47.4 (40.8–50.4)	47.9 (43.5–53)	51.6 (45.6–55.7)
African American (%)	90	90	79	100	91
Male (%)	52	42	43	64	64
High school education (%)	41	39	50	33	41
Ever married (%)	39	42	32	41	41
Income, yearly <$5000* (%)	78	83	89	71	64
Had insurance* (%)	85	71	79	96	100
Homeless* (%)	8	6	14	4	9
Substance use variables*					
Cigarette use (%)	83	77	75	93	91
Alcohol use (%)	65	61	61	68	73
Marijuana use (%)	25	39	21	14	23
Heroin or cocaine use (%)	61	55	46	89	55
Cocaine use (%)	46	42	36	64	41
Heroin use (%)	46	52	36	57	36
Speedball (%)	24	21	15	38	23
Clinical variables					
Depressive symptoms (CESD > 23) (%)	24	29	25	21	18
Methadone treatment* (%)	24	16	21	21	41
Hepatitis C virus seropositive (%)	86	84	79	89	95
HIV positive (%)^†^	59	16	32	100	100
Median CD4 (IQR)^†^	360.5 (239–529)	451 (380–529)	328 (242–404)	327.5 (244–437)	414.5 (166–612)
HIV viral load > 500 copies/mL (%)^†^	55	60	78	61	36

*Represents self-reported exposure during the prior 6 months.

^†^HIV+ status was an inclusion criteria for Trials 3 and 4; CD4 and viral load tested on HIV-positive participants only.

**Table 2:** Feasibility measures by trial*.

Measure	All trials (*N* = 109)	Trial 1 (*N* = 31)	Trial 2 (*N* = 28)	Trial 3 (*N* = 28)	Trial 4 (*N* = 22)
Total days followup	3047	919	817	757	554
Median days of followup (IQR)	28 (26–29)	29 (28–32)	29 (27–33)	28 (25–28)	27 (26–28)
Daily EMA responses					
Random-prompts delivered (*N*)	11181	3462	2317	2654	2748
Random-prompts delivered (%)	78%	80%	60%	73%	98%
Random-prompts answered (*N*)	8655	2816	1940	1985	1914
Random-prompts answered (%)	77%	81%	84%	75%	70%
≥80% response	46%	58%	61%	32%	27%
≥60% response	86%	94%	93%	79%	78%
Random-prompts answered (daily mean)	2.84	3.06	2.37	2.62	3.45
Drug using and craving events					
Participant-initiated events (*N*)	2798	656	836	425	881
Median events initiated (IQR)	11 (3–24)	20 (6–26)	12.5 (1–36)	9 (4–20)	6.5 (1–15)
Craving events initiated (IQR)	8 (5–14)	9 (3–17)	5.5 (0–23)	4 (1–10)	4 (1–11)
Using events initiated (IQR)	3 (0–9)	4 (0–12)	1 (0–11)	4 (1–8)	0 (0–3)
Device retention					
Device loss (1 per × days)	190.4	306.3	204.3	108.1	277.0
PDAs/smartphones issued	140	38	33	43	26
PDAs/smartphones lost	15	3	4	6	2
GPS issued	61	0	30	31	0
GPS lost	1	0	0	1	0
Participant incentives					
Total $ paid	$46,579.00	$12,186.00	$11,986.00	$12,737.00	$9,670.00
Cost/participant	$427.33	$393.10	$428.07	$454.89	$439.55
Cost/person-day	$15.29	$13.26	$14.67	$16.83	$17.45

*During one-month followup.

**Table 3:** Participant acceptability.

Question	Trials 2–4 (*N* = 55)
*In general, how easy is it to use the PDA/phone? *	
Very easy	73%
Easy	25%
Difficult	2%
*What do you think about the number of times that your PDA/phone beeps every day? *	
Not enough	20%
Just right	73%
A little too much	7%
*Do the questions on the PDA/phone make sense to you? *	
All make sense	56%
Most make sense	33%
Some do not make sense	11%
*Does carrying the device(s) make you behave differently than if you didn't have it? *	
Yes	29%
No	71%
*Do you feel comfortable carrying the GPS unit? *	
Extremely comfortable	53%
Mostly comfortable	31%
Somewhat comfortable	15%
Not too comfortable	2%
*Do you feel confident that the information collected by the GPS unit will only be seen by researchers and not used against you? *	
Extremely confident	69%
Mostly confident	22%
Somewhat confident	9%
Not Too confident	0%
Did you have any problems with the GPS unit?*	
Yes	15%
No	85%
How easy is it to carry both the GPS unit and PDA at the same time?*	
Very easy	68%
Easy	24%
Difficult	9%

*GPS survey questions completed from Trials 2 and 3 only (*N* = 34).

**Table 4:** Sociodemographic, behavioral, clinical, and trial characteristics associated with ≥80% weekly EMA response rates.

Variable	Model A			Model B			Model C			Model D		
	OR	95% CI	*P* value	OR	95% CI	*P* value	OR	95% CI	*P* value	OR	95% CI	*P* value
Sociodemographic variables												
Age, 50+	0.99	(0.55–1.79)	0.974	1.22	(0.66–2.25)	0.527	1.03	(0.56–1.89)	0.918	1.14	(0.61–2.10)	0.686
Male	1.4	(0.80–2.46)	0.239	1.21	(0.68–2.15)	0.516	1.34	(0.76–2.37)	0.305	1.24	(0.70–2.20)	0.463
African American	0.81	(0.32–2.07)	0.665	1.23	(0.45–3.38)	0.689	0.86	(0.33–2.22)	0.749	1.18	(0.42–3.29)	0.754
High school education	**2.07**	**(1.15–3.67)**	**0.012**	**2.06**	**(1.15–3.66)**	**0.015**	**1.82**	**(1.03–3.25)**	**0.040**	**1.94**	**(1.08–3.47)**	**0.026**
Used heroin or cocaine^†^	0.89	(0.65–1.42)	0.633	0.85	(0.51–1.40)	0.523	0.79	(0.49–1.29)	0.352	0.84	(0.51–1.40)	0.503
Used heroin or cocaine, past 6 months	1.18	(0.64–2.15)	0.599	1.72	(0.89–3.33)	0.106	1.19	(0.65–2.18)	0.576	1.73	(0.88–3.44)	0.114
Viral load												
HIV negative								Reference			Reference	
Viral load undetectable							**0.50**	**(0.26–1.00)**	**0.046**	1.61	(0.56–4.63)	0.378
Viral load detectable							**0.31**	**(0.16–0.60)**	**0.000**	0.89	(0.34–2.35)	0.821
Week												
1					Reference						Reference	
2				1.10	(0.67–1.81)	0.712				1.09	(0.66–1.82)	0.724
3				1.43	(0.80–2.54)	0.223				1.43	(0.80–2.56)	0.225
4				1.65	(0.90–3.03)	0.103				1.66	(0.90–3.06)	0.105
Trial												
1					Reference						Reference	
2				1.19	(0.55–2.53)	0.661				1.18	(0.56–2.52)	0.665
3				**0.24**	**(0.11–0.53)**	**0.000**				**0.22**	**(0.07–0.69)**	**0.009**
4				**0.38**	**(0.17–0.85)**	**0.019**				**0.29**	**(0.09–0.92)**	**0.035**

^†^Used heroin or cocaine that week, reported by EMA.

Bold indicates statistically significant associations with *P* value < 0.05.
